# Microarray meta-analysis database (M^2^DB): a uniformly pre-processed, quality controlled, and manually curated human clinical microarray database

**DOI:** 10.1186/1471-2105-11-421

**Published:** 2010-08-10

**Authors:** Wei-Chung Cheng, Min-Lung Tsai, Cheng-Wei Chang, Ching-Lung Huang, Chaang-Ray Chen, Wun-Yi Shu, Yun-Shien Lee, Tzu-Hao Wang, Ji-Hong Hong, Chia-Yang Li, Ian C Hsu

**Affiliations:** 1 Department of Biomedical Engineering and Environmental Sciences, National Tsing Hua University, No. 101, Section 2, Kuang-Fu Road, Hsinchu, 300, Taiwan; 2 Institute of Athletics, National Taiwan Sport University, No. 16, Section 1, Shuan-Shih Road, Taichung, 404, Taiwan; 3 Institute of Statistics, National Tsing Hua University, No. 101, Section 2, Kuang-Fu Road, Hsinchu, 300, Taiwan; 4 Department of Biotechnology, Ming Chuan University, 5 De Ming Rd., Gui Shan District, Taoyuan, 333, Taiwan; 5 Genomic Medicine Research Core Laboratory, Chang Gung Memorial Hospital, No.5, Fuxing St., Taoyuan, 333, Taiwan; 6 Department of Obstetrics and Gynecology, Lin-Kou Medical Center, Chang Gung Memorial Hospital and Chang Gung University, No.5, Fuxing St., Taoyuan, 333, Taiwan; 7 Department of Radiation Oncology, Chang Gung Memorial Hospital, No.5, Fuxing St., Taoyuan, 333, Taiwan; 8 Department of Medical Imaging and Radiological Science, Chang Gung University, No.259 Wen-Hwa 1st Road, Kwei-Shan, Taoyuan, 333, Taiwan

## Abstract

**Background:**

Over the past decade, gene expression microarray studies have greatly expanded our knowledge of genetic mechanisms of human diseases. Meta-analysis of substantial amounts of accumulated data, by integrating valuable information from multiple studies, is becoming more important in microarray research. However, collecting data of special interest from public microarray repositories often present major practical problems. Moreover, including low-quality data may significantly reduce meta-analysis efficiency.

**Results:**

M^2^DB is a human curated microarray database designed for easy querying, based on clinical information and for interactive retrieval of either raw or uniformly pre-processed data, along with a set of quality-control metrics. The database contains more than 10,000 previously published Affymetrix GeneChip arrays, performed using human clinical specimens. M^2^DB allows online querying according to a flexible combination of five clinical annotations describing disease state and sampling location. These annotations were manually curated by controlled vocabularies, based on information obtained from GEO, ArrayExpress, and published papers. For array-based assessment control, the online query provides sets of QC metrics, generated using three available QC algorithms. Arrays with poor data quality can easily be excluded from the query interface. The query provides values from two algorithms for gene-based filtering, and raw data and three kinds of pre-processed data for downloading.

**Conclusion:**

M^2^DB utilizes a user-friendly interface for QC parameters, sample clinical annotations, and data formats to help users obtain clinical metadata. This database provides a lower entry threshold and an integrated process of meta-analysis. We hope that this research will promote further evolution of microarray meta-analysis.

## Background

Rapid accumulation of vast amounts of microarray data in public databases like Gene Expression Omnibus (GEO) [1] and ArrayExpress [2] over the past few years has now made it possible to retrieve, integrate, and compare microarray results from many datasets [3,4]. Research has used meta-analysis of microarray results by integrating data from multiple independent studies to successfully identify novel prognosis and diagnosis signatures for cancer and other diseases [5-8]. Microarray meta-analysis involves a systematic search for suitable datasets in retrieval, filtering, re-processing, integration, and analysis. The entire process is complex, laborious, and time-consuming [4]. In an effort to disentangle the complexity of microarray meta-analysis studies, Ramasamy et al. addressed several key issues [4]. However, as noted by these authors, obstacles and challenges remain.

First, identifying suitable studies for meta-analysis is a time-consuming process because experimental information is often stored in a free-text format in a data warehouse. Although most microarray repositories have adopted the Minimum Information about a Microarray Experiment (MIAME) [9] standard, consistent formats and terminologies for annotating experiments and samples are not specified. The completeness and accuracy of information largely depend on the meticulousness of authors, and this issue constitutes a major challenge for microarray meta-analysis.

Second, re-processing raw data is of great importance for integrating data from multiple datasets [4,10,11], but raw data are not always available. As cautioned by Ochsner et al., fewer than 50% of all microarray studies published in the twenty top-ranked journals during 2007 resulted in depositing datasets into microarray data repositories [12]. Only some of the deposited datasets provide raw intensity data. Taking the Affymetrix HG-U133A platform as an example, only 44% of the samples submitted to the GEO contain raw data files [10].

Third, using pre-processed data by different algorithms will introduce variations into the results of meta-analysis [4,13]. Different datasets typically use different normalization methods, and therefore data downloaded from different sets of experiments are unlikely to be directly comparable. These data are unsuitable for meta-analysis and may produce non-combinable results [4,14]. As suggested by Ramasamy et al., even for studies conducted using the same microarray platform, the raw data should be uniformly pre-processed and normalized using the same algorithm to remove systematic biases for all tested datasets [4].

Fourth, several investigators have suggested considering data quality within the context of microarray meta-analysis [4,11,13]. Recent studies conducted by the MicroArray Quality Control (MAQC) consortium and others have demonstrated that good laboratory proficiency and the resulting improved data quality significantly enhance inter-laboratory and inter-platform reproducibility [15-17]. Moreover, including potential outliers in meta-analysis reduces normalization efficiency, especially when using small datasets for pre-processing [11]. Therefore, it is important to identify and eliminate poor-quality data before the pre-processing step [4,10]. However, quality assessment does not accompany microarray data retrieved from public repositories. Therefore, extra efforts are needed to determine the quality of retrieved microarray data.

A number of databases or web servers have recently been developed to tackle these problems. For example, Celsius [18] is a data warehouse that collects Affymetrix CEL files and seven kinds of pre-processed metadata. CleanEX [19] re-annotates microarray datasets with MeSH terms to facilitate the data-retrieval process. MaRe [20] and GEOmetadb [21] provide tools to facilitate the search and retrieval of data from GEO or ArrayExpress. The M^3D ^[14] has collected Affymetrix microarray data and provides manually curated experimental conditions, and uniformly normalized microarray data on three microbial species for download. Oncomine [22] has extensively collected, annotated, and standardized human cancer arrays for various platforms. GeneSapiens [23] re-annotates the samples, applies quantile normalization, and offers gene-based scatterplot/correlations between pairs of genes across tissues in its website. However, depending on the aim and scope of these studies, the problems listed above have only been partially resolved.

This study develops M^2^DB, an expert curated database, to solve microarray data retrieval, annotation, pre-processing, and quality-assessment problems. M^2^DB contains more than 10,000 previously published Affymetrix array data, re-annotated with controlled vocabularies from ontologies (most from NCI Thesaurus), according to available clinical information. Samples of interest can be easily queried, based on five clinical annotations: "Disease State," "Disease State Suppl.," "Disease Location," "Organism Part," and "Organism Part Subtype." Raw data were retrieved from HG-U133A and HG-U133 plus 2.0 and were uniformly pre-processed using Affymetrix Microarray Suite 5 (MAS5) [24], robust multi-chip average (RMA) [25], and GC-robust multi-chip average (GCRMA) [26]. Quality-control assessment reports, for array-base filtering, generated using SimpleAffy [27], Mahalanobis Distance Quality Control (MDQC) [28], and Parametric MultiVariate Outlier labeling (PMVO) [29], packages of Bioconductor [30], were provided for all samples. Arrays with low-quality measurements can be easily excluded from the M^2^DB web query interface. MAS5call and labeling efficiency values (LEVs) [31] are supplied for gene-based filtering for further analysis. The many features offered by M^2^DB efficiently facilitate the search and retrieval process, as well as ensuring the reliability of human clinical microarray metadata. In summary, M^2^DB provides human curated annotations, raw data, uniformly preprocessed data, and sets of QC metrics, and significantly improves the quality and comparability of microarray metadata generated by different laboratories.

## Construction and content

**Figure 1:**
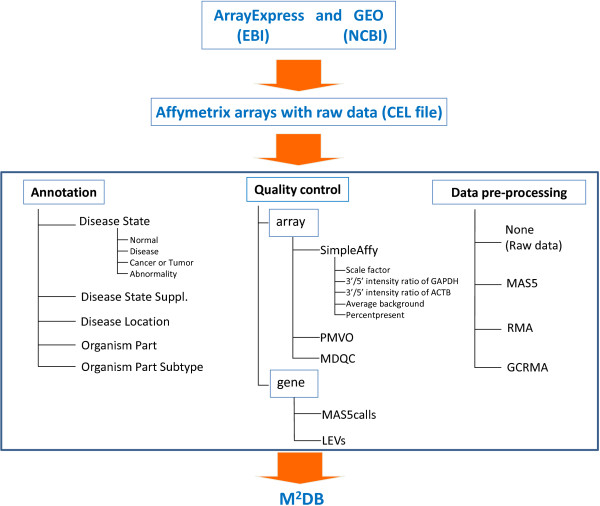
**Schematic process diagram of M^2^DB**. Affymetrix GeneChip HG-U133A and HG-U133 Plus 2.0 arrays with CEL files are downloaded from GEO and ArrayExpress. Then, raw data was pre-processed using MAS5, RMA, and GCRMA. All arrays were annotated into five annotations via manual curation. Array quality assessed by SimpleAffy, PMVO, and MDQC packages in Bioconductor. MAS5call and labeling efficiency values (LEVs) are supplied for gene-based filtering for further analysis. Finally, all information was stored in M^2^DB.

As illustrated in Figure 1, datasets for human studies were collected from public repositories. After the completion of sample annotation, quality assessment, and data pre-processing, the data were stored in the M^2^DB server.

### Dataset collection and pre-processing

Raw intensity data (CEL files) generated using Affymetrix HG-U133A and HG-U133 plus 2.0 platforms were retrieved from GEO and ArrayExpress. Arrays performed using samples other than human clinical specimens, such as cell lines, primary cells, and transformed cells, were excluded. In addition, datasets without links to publications were also excluded except Expression Project for Oncology (expO) (GSE2109). A total of 69% of published arrays which contain raw data were performed by human clinical samples. The other 31% of arrays were hybridized to cell lines, primary culture cells, transformed cells, etc. About 8% of clinical arrays were uploaded to repositories more than once. We removed the redundant arrays according to the generation date of CEL files. Eventually, M^2^DB contains more than 10,000 Affymetrix GeneChip arrays from 192 experiments in ArrayExpress (158 out of 192 experiments also belong to GEO). All microarray raw data were pre-processed using three different algorithms: MAS5, RMA, and GCRMA as implemented in the Bioconductor packages. RMA and GCRMA processed data on a multi-array basis; therefore all arrays of the same platform were uniformly pre-processed to reduce variance.

The Affymetrix microarray system is recognized as naturally suited for meta-analysis [32] and was used as the only microarray system in M^2^DB, based on several factors. First, the Affymetrix platform provides a consistent and reliable system with a high level of reproducibility [10,14,33]. Second, mapping probes to genes using datasets on different platforms is a complex process [4]. Using datasets originating from the same platform circumvents this problem. Third, the single-channel design enables between-chip comparison without a common reference for all arrays [14]. Fourth, many pre-processing, normalization, and QC algorithms are readily available for the Affymetrix platform. Fifth, Affymetrix is the most popular commercial microarray platform, and a very large number of Affymetrix microarray datasets have been deposited into public microarray repositories [34]. On the other hand, there are several obstacles in integrating data from different platforms (including Affymetrix and non-Affymetrix), such as various data processing methods, complex probe-to-gene relationships, and difficulty in comparing results [17]. Moreover, the strategy of M^2^DB is providing relative "pure" data to reduce the variance of data processing in meta-analysis. For these reasons, we excluded non-Affymetrix platforms from M^2^DB.

### Sample annotation

To ensure annotation consistency and make the retrieval process more efficient, clinical information for each sample was manually curated, based on data obtained from GEO, ArrayExpress, and published papers. As shown in Figure 1, each sample was re-annotated with five clinical characteristics: Disease State, Disease State Suppl., Disease Location, Organism Part, and Organism Part Subtype. Organism Part and Organism Part Subtype describe the sample location. Disease State, Disease State Suppl., and Disease Location describe the physiological state and disease information of individuals. "Organism Part" describes the anatomical location of a sample, such as tissue, organ, blood, or body part. "Organism Part Subtype" is an additional annotation for "Organism Part," containing information such as cell types or specified regions in an organ (for example, Organism Part: heart; Organism Part Subtype: left ventricular). "Disease State" simply classifies samples into four categories: Normal, Cancer or Tumor, Disease, and Abnormality. "Normal" means specimens were collected from apparently healthy individuals without signs of disease. "Cancer or Tumor" specifies that the sample was collected from a cancerous tissue. "Disease" describes that the sample was collected from a diseased site or one under the influence of disease. "Abnormality" means the specimen was collected from an apparently healthy individual who was under the influence of chemical agents, such as alcohol, or was classified as having a metabolic syndrome, such as abnormal glucose tolerance. These annotations are designed to help users find the required samples quickly and easily. "Disease State Suppl." contains supplementary information for "Disease State," for example, the disease name for a "Disease" specimen or the reasons why a sample is annotated as "Abnormality." "Disease Location" specifies the anatomical location of a disease or the primary site of a cancer. In most cases, clinical samples were obtained from the diseased organ, and therefore the organism part is the same as the disease location. However, when samples were obtained from a tissue other than the diseased organ, the organism part is different from the disease location.

These five characteristics were derived from MGED ontology, but some were modified for easier querying. In order to obtain an accurate clinical annotation from the free-text descriptions in GEO, ArrayExpress, and related papers, terms used in annotations are controlled vocabularies from existing ontologies (besides the terms of Disease State Suppl. in Abnormality of Disease State). This research manually extracted relative information from the free-text description of each sample, then identified accurate terms and unified the synonyms via the Bioportal [35], Ontology Lookup Service (OLS) [36] and NCI Enterprise Vocabulary Services (EVS). To fit in with the authors' original intention of the adopted papers and provide an accurate clinical annotation from the free-text description, this work adopted several ontologies instead of a single ontology. About 90% of terms are from NCI thesaurus, and 10% from other ontologies such as the Foundation Model of Anatomy (FMA) and the Systematized Nomenclature of Medicine-Clinical Terms (SNOMED-CT). [See Additional file 1 and 2 for each term and its source]. Terms for each sample were identified by a team of six biologists and medical doctors. Therefore, we annotate all samples with these five characteristics. Approximately 5% of clinical arrays were excluded due to incomplete information for sample annotation. Other clinical information, such as sex, age, or ethnicity, was not included in M^2^DB for query because it is frequently not available.

### Data quality control

For array-based quality control, Affymetrix recommends using a set of QC factors to describe hybridization performance and array quality. M^2^DB provides QC metrics generated by three R packages, SimpleAffy, MDQC, and PMVO. SimpleAffy provides a set of QC factors developed based on the Affymetrix QC report. PMVO and MDQC are multivariate approaches that used to evaluate the quality of an array. MDQC examines the "Mahalanobis distance" of its quality attributes from those of other arrays, while PMVO uses parametric multivariate outlier testing using a multivariate Gaussian model.

Five QC factors are included on the query interface for custom definition: scale factor, average background, the 3'/5' intensity ratio of GAPDH, the 3'/5' intensity ratio of beta-actin, and the proportion of probes called present (percentpresent) as provided by SimpleAffy. The default cutoff values were selected according to the recommendations of Affymetrix, SimpleAffy, and Larson et al. [10]: scale factor < 3 fold differences; the 3'/5' intensity ratio of GAPDH < 1.25; the 3'/5' intensity ratio of beta-actin < 3; percentpresent > 10%. These factors are also used for multivariate testing in MDQC and PMVO. Three percentiles, 90, 95, and 99, are provided as selection criteria for respective distributions on the query interface for these two methods.

For gene-based filtering, the database provides MAS5call and LEVs. The MAS5 algorithm supplies the MAS5call, which annotates whether the gene is expressed in specimen. LEV accesses the effect of RNA-labeling efficiency and RNA quality. Research has proven that filtering out genes with highly variable LEV improves the comparability between different laboratories and the homogeneity of gene expression profiles within the same class of specimens [31].

## Utility

### Query interface

**Figure 2:**
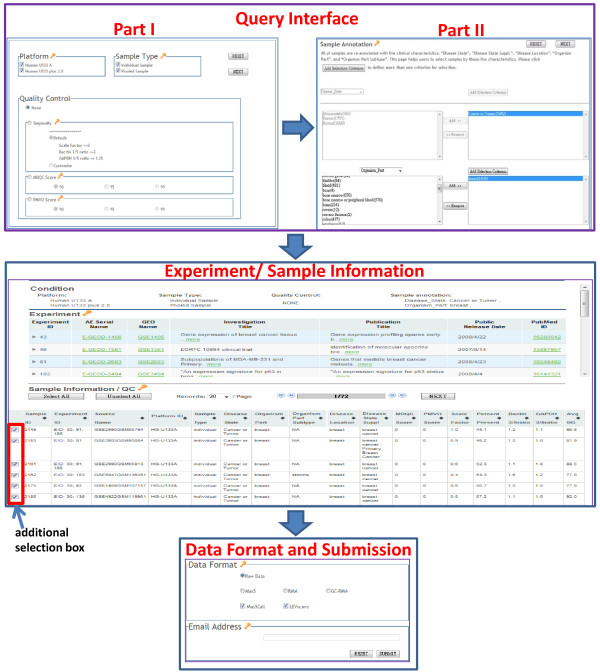
**Web query interface of M^2^DB**. M^2^DB Web interface consists of three parts: 1). Sample selection according to sets of QC metrics and manually curated annotation, 2). The display of Experiment/Sample information, where the "additional selection box" enables to make further selection, and 3). Data Format and Submission panel.

As shown in Figure 2, the M^2^DB web query interface consists of two parts. Part I provides "sample type," "platform," and "quality control" criteria for selection. "Sample type" indicates whether the RNA samples for hybridization are "individual," from a single individual, or "pooled," from multiple individuals. Only two platforms, HU133A and HU133 plus 2.0, are supported in M^2^DB, and users can choose either one or both. The "quality control" option provides SimpleAffy, PMVO, and MDQC QC factors. For SimpleAffy, users can choose the default value or define a customized value for each QC factor. For PMVO and MDQC, three distribution percentiles are offered in their respective algorithms as QC thresholds.

Part II provides a "flexible combination selection" of five clinical annotations for samples which passed the criteria in Part I. Users can define from one to five annotations for query. The types of annotation and their quantities can be visualized in real time according to users' combined selections. This combination selection helps users quickly reduce the number of items submitted for query. For example, there are more than 250 items in "Disease State Suppl." By applying the combination of "Cancer or Tumor" in "Disease State" and "uterine cervix" in "Disease Location", the number of items submitted for selection in "Disease State Suppl." reduces to less than five. On the other hand, users can define different combinations according to their demand. For example, users can select 222 tumors collected from the lung by applying the combination of "Cancer or Tumor" in "Disease State" and "lung" in "Organism Part". When adding the criterion: "lung" in "Disease Location", there are only 215/222 samples classified into lung cancer. In this case, 7/222 samples are classified into lung metastasis.

### Experiment/Sample information

The experiment information table contains simple descriptions of experiments and hyperlinks to the GEO, ArrayExpress, and PubMed web sites to obtain more complete information. The sample information table contains complete clinical annotations and QC factors from SimpleAffy, PMVO, and MDQC for each sample. Moreover, the database provides an "additional selection box" for each sample to enable making further selections. Users have an additional option to decide whether a sample is suitable for further analysis according to the complete information in the table.

### Data format and submission

After confirming the selected samples, users can define which type of data to download. M^2^DB offers four data types: one is raw data (CEL files), and the other three are normalized data (RMA, GCRMA, and MAS5). The database also provides MAS5call and LEVs as gene-base filtering. After entering an email address, the user submits the job to the server. The user receives two email alerts. One is a confirmation email informing the user that the job has been successfully submitted and is being processed. The other email with a download link is sent when the job is completed.

## Discussion

To quickly and easily query samples, annotation categories adopt a flat list instead of a tree structure. For example, only two annotations, the Organism Part and the Organism Part Subtype, are used to describe the sample location in anatomic position. This choice is motivated by the increased complexity of the ontological tree structure in our web design. Additionally, the Organism Part is only used to describe the sample location in MGED ontology. In M^2^DB, we created the Organism Part Subtype to assist users to define the sample location. For example, T lymphocyte samples can be derived from blood, bone marrow, or umbilical cord blood in the database. The two annotations, Organism Part and Organism Part Subtype, can be more accurate, efficient, and less complicated to define the sample location. According to our annotation categories, users can easily and quickly find samples defined in the selection via our web query interface. It provides instantaneous visualization and selective combination (up to five criteria) of the various quantities and types of items selected.

Detailed descriptions of experimental parameters and sample clinical information are necessary to make the metadata fully interpretable. However, complete descriptions are frequently not available or only partially available in either microarray repositories or published papers. Accordingly, in M^2^DB, the authors supplied elementary annotations that were manually curated according to free-text descriptions of the collected experiments. If researchers require further clinical information for advanced analysis, support from the authors of original published papers will be necessary. The authors therefore urge public microarray repositories to request microarray researchers for more detailed information, such as sex, age, disease-free survival...etc. This would greatly encourage microarray meta-analysis across different experiments.

The uniform pre-processing eliminates the technical variance of data transformation, such as background correction, probe-set summarization, and normalization. Gagarin et al. demonstrated that two different summarizations of the same data may produce differential expression gene (DEG) lists that are only 30% concordant [37]. However, laboratory-to-laboratory variation is hard to eliminate, even if adopting the same data transformation process. Yang et al. carried out a study in which a common set of RNA sample was performed five times in four different laboratories using Affymetrix GeneChip arrays. Significant discrepancies exist in intensity profiles and DEG lists across laboratories [38], resulting in intrinsic variance for meta-analysis studies. There are several statistical algorithms developed to relieve this problem [39-42]. Microarray analysis websites, for example ArrayMining [43], also provide cross studies/platforms normalization. Another way to alleviate laboratory-to-laboratory variance is by removing poor quality arrays. Several studies have emphasized the importance of QC for integrative microarray studies [4,10,11,13]. Owzar et al. proved that removing the outlier arrays could relieve batch effect [11]. Ramasamy et al. suggested array quality control as one of the key issues of microarray meta-analysis studies [4].

Housekeeping genes have been used for normalization in gene expression analysis, such as quantitative RT-PCR, northern blotting, and gene expression microarray [44-47]. Furthermore, the expression variation of housekeeping genes between arrays has been used to evaluate the effectiveness of normalization methods [48]. We had used the expression variation of housekeeping genes to examine the effect of array quality control. HU133A arrays performed by normal skeleton muscle in M^2^DB were selected for the analysis. After submitting these clinical annotations for query, forty-nine samples from seven different datasets were identified by M^2^DB. The expression variation of each housekeeping gene is presented as C.V. of intensity as shown in the Additional file 3. In general, the expression variation of the housekeeping genes was reduced when one of the array-based QC methods was applied. These results indicate that applying anyone of the array-based QC methods effectively excludes arrays with poor quality and reduces laboratory- to-laboratory variance in the microarray meta-analysis.

M^2^DB can be used by researchers to collect metadata for the following purposes: 1) Searching for biomarkers of prognosis or disease [49-51]. 2) Using metadata to validate their own results. For example, according to gene expression pattern derived from 28 patients, Vachani et al. identified a panel of ten genes to accurately distinguish two tumor types; this set of marker genes was validated by 134 individuals collected from four independent previously published Affymetrix datasets [52]. 3) Integrating with their own datasets to increase sample size. For example, Lu et al. applied a meta-analysis of datasets including their own samples and five experimental data collected from other microarray studies [53]. Furthermore, for clinical studies, collecting normal samples is a major difficulty. M^2^DB includes more than 1,800 normal samples from healthy individuals without diseases, abnormalities, or treatments according to the descriptions of the experiments. These data from normal samples can help researchers discover and address the differences between normal and diseased (abnormal) specimens by cross-comparing different datasets.

Many public microarray web servers have provided analysis tools such as differential expression, clustering, and supervised classification. Thus, M^2^DB does not put extra effort into constructing online analysis tools. Users can directly upload the M^2^DB's results to those analysis web servers, for example Expression Profiler [54], GEPAS [55], EzArray [56], or ArrayMining [43]. Users with advanced knowledge and skills in data analysis may find it is more feasible to download raw data files (CEL files) and QC metrics to local computers or to transfer them to public analysis web servers, such as WebArrayDB [57], CARMAweb [58], Expression Profiler [54], GEPAS [55], and EzArray [56], which allow user upload CEL files, for more advanced meta-analysis.

MIAME 2.0 now requests authors to deposit their raw data files in public microarray depositories. This policy will greatly help in data integration and meta-analysis. M^2^DB is updated periodically to incorporate new experiments which provide raw intensity data. Newly incorporated microarray data will be re-annotated. It took six researchers about one month to curate ~20,000 arrays (including clinical and non-clinical arrays) and to annotate clinical arrays into five clinical characteristics. Finally, we selected 10,202 arrays into M^2^DB. In the future, when expending the dataset, the needed time will be proportional to the amount of new arrays. In addition, the entire set of raw data will be uniformly re-processed using normalization as well as QC algorithms when adding new chips into M^2^DB.

## Conclusions

This research develops M^2^DB to facilitate the search and retrieval process, as well as to ensure the reliability of human clinical microarray metadata. Providing raw data, uniformly pre-processed data, and several sets of QC metrics, M^2^DB can be used to significantly improve the quality and comparability of microarray metadata generated by different laboratories. The manually curated annotations with the "flexible combination selection" relieve time-consuming searching and help researchers easily find the clinical expression data they need. M^2^DB provides a lower entry threshold and an integrated process of meta-analysis. We hope that this database will promote further evolution of microarray meta-analysis.

## Availability and requirements

The web-application is freely accessible at http://metadb.bmes.nthu.edu.tw/m2db/.

## Authors' contributions

WC, MT, and IH wrote the manuscript. WC, CH, and CWC collected the datasets. WC, MT, TW, JH, CL, and CWC carried out sample annotation. YL and WS performed QC and statistical analysis. CRC and CWC carried out the data pre-process and database server construction. IH, WS, and CH contributed to the discussion and commented the manuscript. IH financially supported this study. All authors read and approved the final manuscript.

## Supplementary Material

Additional file 1 **Summary list of sample location**. The excel file containing the summary list of sample location for 10202 samples of M^2^DB. Click here for file

Additional file 2 **Summary list of disease information**. The excel file containing the summary list of disease information for 10202 samples of M^2^DB. Click here for file

Additional file 3 **The intensity C.V. of 14 housekeeping genes in normal skeleton muscles**. The expression variation of 14 housekeeping genes in normal skeleton muscles. Click here for file
