# Supplementary material for: Microarray meta-analysis database (M2DB): a uniformly pre-processed, quality controlled, and manually curated human clinical microarray database
Source: BMC Bioinformatics. 2010 Aug 10;11:421. doi: 10.1186/1471-2105-11-421 (PMC2928207; doi:10.1186/1471-2105-11-421)
Supplement: Additional file 3 — The intensity C.V. of 14 housekeeping genes in normal skeleton muscles. The expression variation of 14 housekeeping genes in normal skeleton muscles. [file 1471-2105-11-421-S3.PDF]

Additional File 4

The intensity C.V. of 14 housekeeping genes in normal skeleton muscles

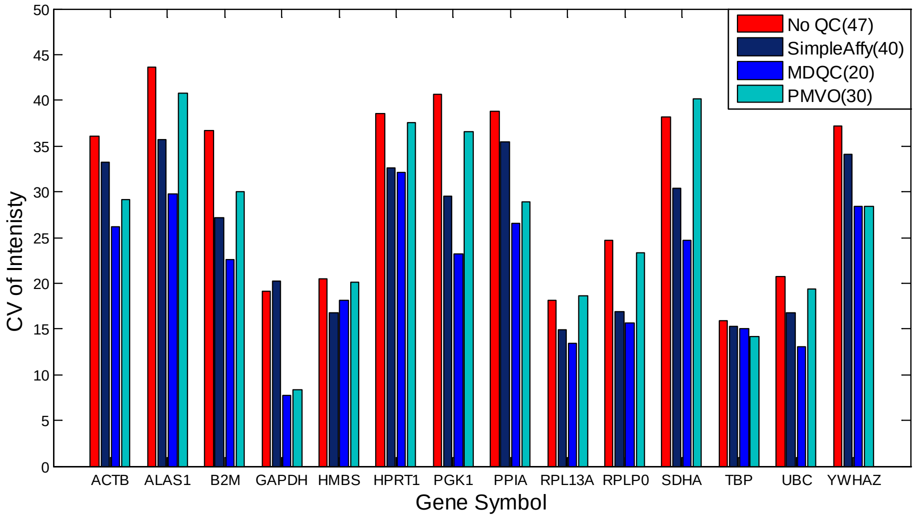

The expression variation of 14 housekeeping genes in normal skeleton muscles is used to evaluate the effect of quality control. The numbers in the legend represent the amounts of array in different quality control algorithms. As multi probe-sets correspond to a single gene, the mean intensity of these probe-sets is used to represent the expression of the gene in the sample. The C.V. of intensity (uniformly processed by RMA without log transformed) is used to represent the variance of gene expression. The default values (scale factor <3, 3'/5' intensity ratio of ACTB and GAPDH < 3 and 1.25, respectively) are used as the threshold in SimpleAffy algorithm. The 90 percentile is the cutoff value for MDQC and PMVO algorithms.
